# Comparative yield of different active TB case finding interventions in a large urban TB project in central Uganda: a descriptive study

**DOI:** 10.4314/ahs.v21i3.3

**Published:** 2021-09

**Authors:** Andrew Kazibwe, Fred Twinomugisha, Joseph Musaazi, Florence Nakaggwa, Disan Lukanga, Phillip Aleu, Timothy Kiyemba, Abel Nkolo, Nicholas Sebuliba Kirirabwa, Debora B Freitas Lopez, Estella Birabwa, Seyoum Dejene, Stella Zawedde-Muyanja

**Affiliations:** 1 The AIDS Support Organization, Kampala, Uganda; 2 USAID/Defeat TB Project, University Research Co. LLC, Kampala, Uganda; 3 The Infectious Diseases Institute, College of Health Sciences, Makerere University, Kampala, Uganda; 4 University Research Co. LLC, Maryland, US; 5 USAID Uganda

**Keywords:** Tuberculosis, screening, community, Uganda

## Abstract

**Introduction:**

Systematic screening for TB among patients presenting to care and among high risk populations is recommended to improve TB case finding. We aimed to describe the comparative yield of three TB screening approaches implemented by a large urban TB project in central Uganda.

**Methods:**

We abstracted data on the screening cascade from 65 health facilities and their surrounding communities (numbers screened, with presumptive TB, receiving a diagnostic test and diagnosed with TB) from the different clinic and community TB registers.

**Results:**

From January 2018 to December 2019, 93,378 (24%) of all patients screened at health facilities had presumptive TB; 77,381 (82.9%) received a diagnostic test and 14,305 (18.5%) were diagnosed with TB. The screening yield (the number of patients diagnosed with TB out of all patients screened) was 0.3% and was three times higher among men than women (0.6% vs 0.2% p<0.01). During targeted community screening interventions, 9874 (21.1%) of all patients screened had presumptive TB; 7034 (71.2%) of these received a diagnostic test and 1699 (24.2%) were diagnosed with TB. The screening yield was higher among men, (3.7% vs 3.3% p<0.01) and highest among children 0–14 (4.8% vs 3.2% p<0.01).

**Conclusion:**

Targeted community TB screening interventions improve access to TB diagnosis for men and children 0–14 years.

## Introduction

During the United Nations (UN) High level meeting held in September 2018, governments committed to find and successfully treat 40 million tuberculosis (TB) patients by 2030.^1^ This commitment aligns with the World Health Organization's END TB strategy goal to reduce global incidence to <20/100,000 by 2030^2^. In order to do this, the WHO recommends the engagement of all care providers in systematic screening for TB^3^.

The WHO defines systematic screening as the regular identification of people with suspected active TB, in predetermined target groups, using tests or procedures that can be applied rapidly. Among those with active TB, diagnosis is then made using diagnostic tests with high specificity^3^,^4^. In this context, systematic screening is predominantly provider-initiated and may target people in the community who have not yet sought healthcare services or people who have presented to public health facilities but do not have or do not recognize TB signs and symptoms. Systematic screening may also be targeted to specific patient populations with comorbidities, e.g., HIV and Diabetes Mellitus that increase their likelihood of having TB. Compared to passive TB case-finding, which looks for TB among people actively seeking care due to symptoms compatible with TB^5^, systematic screening increases the number of patients initiated on TB treatment and results in earlier case-finding. Randomized community trials in Brazil and Ethiopia investigating the effect of systemic screening on the number of TB cases found showed an increase in case-finding in intervention communities compared to control communities^6^,^7^. Studies comparing delays to treatment initiation or extent of disease at presentation between those identified through systematic screening and those identified through passive case finding found that patients identified through systematic screening had shorter duration of symptoms^8^,^9^, lower grades of smear positivity8 and were less likely to have severe chest X-ray features such as cavitation^10^.

Uganda remains among the high TB/HIV burden countries in the world which targets to find and treat at least 80% of all incident TB cases by 2024^11^. The Ministry of Health's National Tuberculosis and Leprosy Program (NTLP) aims to achieve this target by emphasizing the systematic screening for TB among all patients presenting for care at health facilities; and among selected high risk populations including household and close contacts of patients diagnosed wth TB and persons living in congested and congregational settings e.g., slum dwellings, correctional facilities and schools^11^. However, because systematic screening is a resource intensive exercise with variable yield depending on the TB prevalence of the population where it is carried out, it is important to focus on the areas with the highest yield. We aimed to describe the yield of different systematic screening efforts in order to guide TB case-finding efforts in Uganda.

## Methods

### Study setting

Kampala, Mukono, and Wakiso are three urban districts situated in central Uganda with a combined mid-year population of 5.1 million in 2019^12^. The three districts have an overall estimated HIV prevalence of 6.2%, higher in women (7.6%) than men (4.7%)^12^ and an estimated TB prevalence of 231/100,000 population, which is four times higher among men than women (734/100,000 population vs 179 / 100,000 population)^13^.

Similar to many urban districts in sub-Saharan Africa, a considerable proportion of the population (54%) lives in slum dwellings with high population densities, poor ventilation, and high TB transmission^12^. Majority of the population seek care in 149 public health facilities which register about 2.5 million patient visits each year^14^. Healthcare in the public health system is delivered in a tiered system from primary to tertiary levels of care. The primary care system consists of Health Center (HC) IIs delivering preventive, promotive and outreach curative services; Health Center IIIs which deliver, in addition to the above, maternity, inpatient health and laboratory services; Health Center IVs which deliver, on top of all the services delivered at HC IIs and IIIs, emergency surgery and transfusion services. The tertiary care system consists of hospitals which provide in addition to the services at primary care facilities, in-service training, consultation and specialized services^15^

In 65 of these public health facilities, the Defeat TB project, funded by the United States Agency for International Development (USAID), partners with the Ministry of Health's NTLP to improve TB screening, diagnostic, and treatment services (Table 1). During the study period, the project supported systematic screening for TB in these health facilities by setting up screening posts at major care-entry points. To reduce the workload on trained healthcare workers brought on by the large numbers of patients screened for TB, the project trained lay workers called “linkage facilitators” to screen all patients for TB, collect sputum, and deliver it to laboratories for TB testing. The project also supported community-based screening by facilitating community healthcare workers to systematically screen for TB among household and close contacts of index TB patients and to carry out targeted community screening activities in known TB hotspots e.g., slum dwellings, market places, and taxi/bus terminals. To mitigate TB-related stigma which was recognized as a potential barrier to uptake of community screening, community volunteers are trained to seek consent from index patients prior to household contact tracing and to integrate health education about TB in their community outreaches.

**Table 1:** Structure of the Study Population

Characteristics	Kampala	Mukono	Wakiso
**Population**	1,650,800	682,800	2,735,100
**Sex**			
Male	781700 (47.4%)	330800 (48.4%)	1296500 (47.4%)
Female	869100 (52.6%)	352000 (51.6%)	1438600 (52.6%)
**Residence**			
Rural	-	434062 (72.7%)	814517 (40.8%)
Urban	1507080 (100%)	162742 (27.3%)	1182901 (59.2%)
**Residence type**			
Slum Dwelling	807795 (53.6%)	87230 (53.6%)	634035 (53.6%)
Not slum dwelling	699285 (46.4%)	75512 (46.4%)	548866 (46.4%)
**HIV Prevalence**	6.9%	7.6%	8.0%
**Health facilities**			
HC II	3 (12.0%)	-	1 (4.0%)
HC III	10 (40.0%)	11 (73.3%)	16 (64%)
HC IV	1 (4.0%)	1 (6.7%)	7 (28.0%)
Hospitals	11(44.0%)	3 (20.0%)	1 (4.0%)

*Table 1: Source: 2014 NPHC report, UBOS 2019 population projection* HCII – Health Center II; HC III- Health Center III and HC IV- Health Center IV

Systematic screening in both the health facilities and the community was carried out using a standardized intensified case-finding form which is a four-symptom questionnaire on the presence and duration of symptoms that are possibly suggestive of TB e.g., cough, haemoptysis, weight loss, fever, night sweats^3^,^16^. Patients with cough for two weeks or longer or cough of any duration in addition to other symptoms suggestive of TB were diagnosed with presumptive TB and tested using GeneXpert testing or chest X-ray if they could not produce sputum^16^. A patient was diagnosed with TB if they had a positive GeneXpert test or if they had a negative GeneXpert test but had abnormalities consistent with TB on chest X-ray or if a clinical decision to treat for TB was made by the healthcare worker.

### Data Collection Procedures

This was a retrospective study using data routinely collected from 65 health facilities (15 hospitals, 9 HC IVs and 41 lower primary healthcare facilities). These health facilities notify 75% of all the TB cases in Kampala, Wakiso and Mukono and are prioritized by the Defeat TB project for intensive support. We abstracted data from national TB registers present at the health facilities for the period January 2018 to December 2019. We collected data on the number of patients seen and screened for TB at five selected care-entry points, i.e. outpatient department (OPD), HIV clinics, maternity and child (MCH) clinics, nutrition clinics, and inpatient department (IPD), from the respective daily clinic attendance registers. We abstracted data on the number of presumptive TB cases found, the number investigated for TB and the number diagnosed with TB from the presumptive TB registers at each care-entry point and collaborated it with data from the respective health facility laboratory TB registers. Additional information on patients' age and sex and date seen were extracted from the same registers. In addition, we included data on patients screened for TB during community screening activities implemented by the USAID Defeat TB project. These activities targeted high risk communities e.g. household contacts of patients diagnosed with TB, persons living in slum areas, persons working in open air markets and bus terminals. Data on screening in these communities was collected from the community TB registers. These registers contain data on the number of community screening visits, the number of patients seen at each visit as well as the numbers screened for TB, identified as presumptive TB cases, and diagnosed with TB.

### Data Management and Analysis

Data collected was then captured, cleaned in liaison with data collection teams, and maintained in the project electronic database. To construct the TB diagnostic cascade, we calculated the proportion of presumptive TB identified out of all patients seen at the respective care entry points and in the community. We then calculated the proportion of presumptive TB patients tested for TB out of all presumptive TB cases identified, and finally the proportion of TB cases diagnosed out of all presumptive TB cases tested. We further calculated the screening yield (the proportion of patients diagnosed out of all those screened for TB) for selected care entry points at the health facilities and for two community TB case finding activities (household contact tracing and targeted community TB screening. Data was analyzed using STATA/MP version 14.1.

## Results

From January 2018 to December 2019, there were 6,412,387 patient visits at the 65 health facilities, with the larger proportion of visits (66.6%) made by females. Approximately 28.6% of all patient visits were made by patients below 15 years of age. Although hospitals represented only 23% of the 65 health facilities, they had the highest proportion of patient visits (36.3%). Within the health facility, the highest proportion of patient visits were made to the OPDs (Table 2).

**Table 2:** The TB diagnostic cascade disaggregated by age, sex and care-entry point from 65 health facilities in Kampala, Mukono and Wakiso

Characteristic	Patients Seen	Screened N (%)	Presumptive TB N (%)	Presumptive TB Investigated N (%)	Presumptive TB Diagnosed N (%)	Screening Yield (Overall =0.33%)	
	N=6412387	N=4366277	N=93378	N=77381	N =14305	%	P-value†
**Sex**							
Female	4270011	2963253 (69.4)	48371 (1.6)	39426 (81.5)	6054 (12.5)	0.20	<0.01
Male	2142376	1403024 (65.5)	45007 (3.2)	37955(84.3)	8251 (18.3)	0.59	

**Age** ¶							
0–14	1835139	939622 (51.2)	14231 (1.5)	9038 (63.5)	1508 (10.6)	0.16	<0.01
15+	4577248	3426655 (74.9)	79147 (2.3)	62931 (79.5)	11876 (15.0)	0.35	

**Care Entry point**							
HIV clinic	1234582	1147959 (93.0)	22764 (2.0)	16532 (72.6)	2495 (11.0)	0.22	<0.01
IPD	160951	35479 (22.0)	1390 (3.9)	1625 (116.9)β	426 (30.7)	1.20	
OPD	3189362	2296327(72.0)	66526 (2.9)	57918 (87.1)	11245 (16.9)	0.49	
MCH	1814447	883032 (48.7)	2567(0.3)	1244(48.5)	105 (4.1)	0.01	
Nutrition	13045	3480 (26.7)	131(3.8)	62(47.3)	34(26.0)	0.98	

**Health facility level**							
HC II	236936	205031 (86.5)	4367 (2.1)	3352 (76.8)	607 (13.9)	0.30	<0.01
HC III	2298891	1692319 (73.6)	40502 (2.4)	32845 (81.1)	5078 (12.5)	0.30	
HC IV	1551461	1076202 (69.4)	23337 (2.2)	19447 (83.3)	3919 (16.8)	0.36	
Hospital	2325099	1392725 (59.9)	25172 (1.8)	21737 (86.4)	4701 (18.7)	0.34	

IPD- In Patient Department, OPD- Out Patient Department, MCH- Maternal & Child Health

HCII – Health Center II; HC III- Health Center III and HC IV- Health Center IV

β denotes additional patients for whom a TB diagnostic test is ordered as part of in-patient care but no record was found in the presumptive TB register.

¶ Missing values: Age (Presumptive TB Investigated = 5412[6.9%], Presumptive TB Diagnosed 921[6.4%)])

† Likelihood Ratio P-values obtained from Poisson regression for Rates (that is number of patients diagnosed with TB over the total number screened over the two years studied)

The above data is from different sources (Presumptive and laboratory TB registers)

### The Health Facility TB Diagnostic Cascade

At the health facilities, 68% of all patients seen were screened for TB. The proportion of patients screened for TB ranged from 22% in the IPD to 93% in the HIV clinic. However, regardless of proportions screened for TB, the proportion of presumptive TB cases was between 2% to 4% except in the MCH clinic, where only 0.3% of mothers screened were identified as presumptive TB cases. Overall, 82.9% of all identified presumptive TB cases received a diagnostic test. This proportion ranged from 47% in the nutrition clinics to 100% in the IPD. The highest proportion of patients diagnosed with TB out of all presumptive TB patients was in the IPD (30.6%) and the nutrition clinics (26%), while the lowest was in the MCH clinics (4.1%) (Table 2).

**Figure 1:**
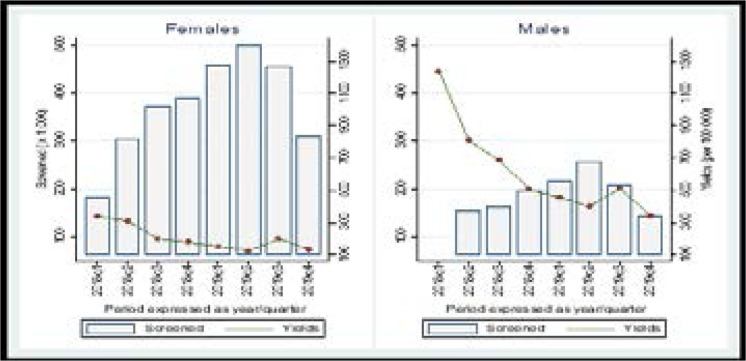
Health facility screening yield (disaggregated by sex)

**Figure 2:**
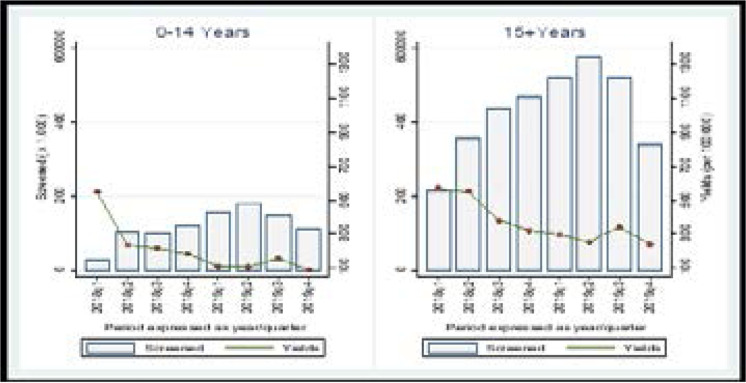
Health facility screening yield (disaggregated by age)

Disaggregated by age and sex; The proportion of patients screened for TB among children 0–14 was lower than that for persons aged 15 and above (51.2% vs 74.9%). Furthermore, the proportion of presumptive TB was higher among persons aged 15 and above (2.3% vs 1.5%). Completion of the diagnostic cascade was better among adults, with 79.5% of adults with presumptive TB receiving a diagnostic test compared to 63.5% of children. The proportion of those diagnosed with TB among all patients tested was also higher for adults as 15% of all those tested for TB were diagnosed with TB compared to 10.6% of children (Table 2; Figure 1 & 2).

Although the number of women screened for TB doubled the number of men screened, the proportion of presumptive TB was twice as high among men (3.2% vs 1.6%,) than women. The proportion of presumptive TB patients getting a diagnostic test was similar for both sexes (84.3% vs 81.5%). However, the proportion of those diagnosed with TB among all patients tested was men higher than women (18.3 vs 12.5%). (Table 2).

### The Community TB Diagnostic Cascade

In total, the project screened 46,469 patients during the targeted community activities. About one in four persons screened was identified as having presumprive TB patients and 71.2% of all presumptive TB received a diagnostic test. About one in five household contacts were identified as presumptive TB patients. This proportion was similar across age-groups and between men and women. About 72% of all household contacts with presumptive TB received a diagnostic test. The proportion diagnosed with TB was higher among contacts 15 years and older than those 0–14 years (14.0% vs 12.6%) and higher among women than men (17.0% vs 15.0%) (Table 3).

**Table 3:** Description of persons from community case-finding activities in Kampala, Mukono and Wakiso

**Patients from targeted community case-finding approaches**							

	**Patients** **Seen** **N (%)**	**Screened** **N (%)**	**Presumptive** **TB** **Identified N** **(%)**	**Presumptive** **TB** **Investigated** **N (%)**	**Presumptive** **TB** **Diagnosed N** **(%)**	**Screening Yield** **(Overall =3.7%)**	
	N=49620	N=46469	N=9874	N=7034	N=1699	%	P-value†

**Age** **(yrs)**							
0–14	14622	13328 (91.2)	3036 (22.78)	2107 (69.4)	640 (21.1)	4.8	<0.01
15+	34998	33141 (94.7)	6838 (20.6)	4927 (72.1)	1059 (15.5)	3.2	

**Gender**							
Male	25690	23901 (93.0)	5197 (21.7)	3813 (73.4)	953 (18.3)	3.3	0.01
Female	23930	22568 (94.3)	4677 (20.7)	3221 (68.9)	746 (16.0)	3.7	

**Household contacts of patients diagnosed with TB**							

	**Patients** **seen**	**Screened, N** **(%)**	**Presumptive** **TB identified** **N (%)**	**Presumptive** **TB** **Investigated** **N (%)**	**Presumptive** **TB Diagnosed** **N (%)**	**Screening Yield** **(Overall =3.6%)**	
	N=63896	N=63896	N=17043	N=12239	N=2328	%	P-value†

**Age** **(yrs)**							
0–14	13831	13831	3686 (26.7)	2325 (63.1)	463 (12.6)	3.3	0.04
15+	50065	50065	13357 (26.7)	9914 (74.2)	1865 (14.0)	3.7	

**Gender**							
Male	30940	30940	9120 (29.5)	6600 (72.4)	1370 (15.0)	4.4	<0.01
Female	32956	32956	7923 (24.0)	5639 (71.2)	958 (17.0)	2.9	

*Table 3: Source: Primary Data*

† Likelihood Ratio P-values obtained from Poisson regression for Rates (that is number of TB patients diagnosed with TB over the total number screened for TB over the two years studied)

### The TB Screening Yield

Within the health facilities, the overall TB screening yield was 0.3% (328/100,000). The screening yield was thrice as high among men (0.6%) (588/100,000) as women (0.2%) (204/100,000), p<0.01 and highest in the IPDs (1.2%) (1200/100,000), p<0.01. The screening yield among persons ≥15 years was twice as high as that among children 0–14 years (0.35% vs 0.16%, p<0.01) (Table 2; Figures 1 and 2).

**Figure 3:**
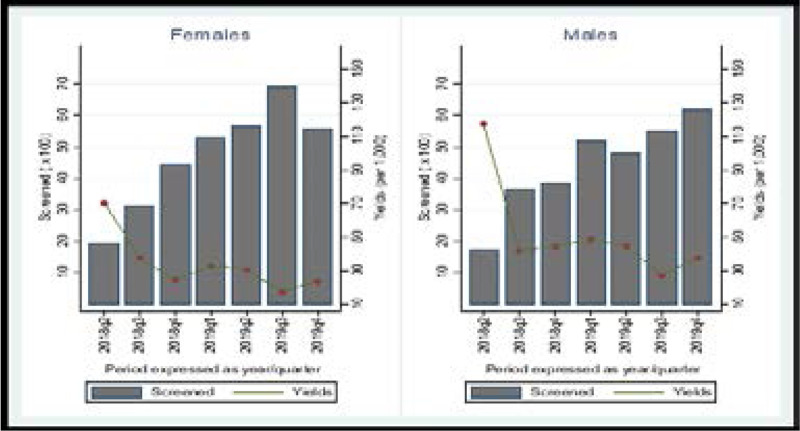
Community screening yield (disaggregated by sex)

Overall, the screening yield was almost ten times higher in targeted community screening interventions than from health facility-based screening (3.7% vs 0.3%). During targeted community screening, the screening yield was also higher among men than women (37/1000 vs 33/1000) (3.7% vs 3.3%, p<0.01) although this was less pronounced than at the health facility (Table 3; Figure 3). The highest screening yield during community interventions was among persons 0–14 years (4.8%). Household contact tracing also resulted in a high screening yield among children 0–14 (3.3%), (33/1000 vs 37/1000). The screening yield among men remained higher during household contact tracing and was 1.5 times higher among men than women (4.4% vs 2.9%) (44/1000 vs 29/1000) (Table 3; Figure 3).

### Initiation of TB treatment

During the period of study, a total of 17,677 patients were started on TB treatment. This included patients diagnosed at both the health facility and the community and those diagnosed referred into public health facilities for TB treatment from private health facilities which have the ability to diagnose TB but are not designated as TB treatment facilities.

## Discussion

In this retrospective study evaluating the different TB case-finding interventions, we analysed data on TB screening and testing from various care-entry points within 65 health facilities and from targeted community case-finding activities. We found that a high proportion of patients completed the TB diagnostic cascade. We also found that the TB screening yield from targeted community interventions, e.g., household contact tracing contributed significantly to TB case finding with high screening yields. In addition, we found that the TB screening yield was higher among men than women but that this ratio decreased as case finding interventions moved from the health facility to the community and finally to the household.

Within the health facility, 68% of all patients seen were screened for TB and 71.2% of all presumptive TB patients received a TB diagnostic test. Although this proportion is below the 90% proposed by the End TB target^17^, it is significantly higher than that reported in previous studies evaluating the TB screening cascade where only 30–50% of presumptive TB patients were evaluated with a diagnostic test^18^–^20^ and can be attributed to the deployment of lay health workers called “linkage facilitators” within the health facilities. These lay health workers expand the health workforce to meet the increased demands that come with screening such large numbers of patients by performing the symptom screen and collecting and transporting sputum samples to the laboratory. The same approach is used during community case finding activities where the community healthcare worker screens patients, collects sputum, and delivers sputum samples to health facility laboratories. The use of lay health workers overcomes previously documented causes of non-completion of the TB screening cascade which include long waiting times at the health facilities and lack of time or transport money for household contacts to visit the health facilities^21^–^23^.

In general, the number of TB patients diagnosed out of all patients screened (screening yield) was twice as high among men. The ratio of the screening yield among men to that among women decreased from 3.0 at the health facility to 1.5 in targeted community interventions to 1.5 in household contact tracing. Although a higher screening yield among men is consistent with findings from several TB prevalence surveys that report hgher prevalence of TB among men^13^,^24^,^25^, it could also be attributed to poor health seeking behaviour resulting in late care seeking. Studies from sub-Saharan African settings including Uganda have found that men are more likely to present to healthcare facilities with advanced HIV disease^26^ and bacteriologically confirmed TB^27^. In our study, poor healthcare seeking can be inferred from the low numbers of men seeking care at the health facilites and from the higher screening yield ratios between men and women at the health facility compared to targeted community interventions.

At the health facilities, the screening yields were highest in the in-patient department and the nutrition clinics. However, the yield from the nutrition clinics could have been improved if a higher proportion of patients received a TB diagnostic test. In our study, poor completion of screening cascade in the nutrition clinics could have been due to failure to produce sputum by children who form the majority of malnourished patients who are children coupled with unavailability of auxiliary diagnostic methods e.g. chest radiography and a lack of skill or confidence among healthcare workers to clinically diagnose TB in children^28^. Nevertheless, our findings empahsize that TB screening among these two populations represents a high yield intervention that can be implemented at minimal additional cost.

Household contact tracing is an entry point for child TB care and serves as an opportunity to carry out timely evaluation of child contacts of index TB patients for treatment of active TB or latent TB infection as appropriate^19^,^20^,^28^. In our study, household contact tracing had an screening yield of 3.3% among children compared to 0.16% among children at the health facility. This emphasizes the need for program managers looking to improve case-finding among children to actively promote contact tracing.

The evaluation had several strengths. First, data from this evaluation was collected prospectively as part of a large ongoing TB implementation project and thus fully reflects programmatic conditions in low resource, high burden settings. Findings from this study are therefore likely to be generalizable to similar settings. Secondly, our cascade analysis adds to a growing body of literature on TB case finding that seeks to account for how patients move through the different steps of the screening, diagnostic and treatment cascade in order to improve case-finding in routine healthcare settings. Our major limitation was that data for this analysis was collected by healthcare workers involved in routine healthcare delivery resulting in some missing data. However, the proportion of missing data for each variable was relatively small and did not significantly affect the integrity of the analysis. Finally, our study did not include a costing element and did not evaluate the impact of community screening on time to TB diagnosis. As community interventions have been previously shown to be resource intensive, evaluating the cost effectiveness of this intervention and the impact on time to TB diagnosis should be the focus of future studies on community TB case finding.

## Conclusion

Targeted community TB screening interventions which focus on areas with high TB incidence have the capacity to improve access to TB diagnosis for men and children 0–14 years. Additional interventions for community-based screening should focus on enabling all presumptive TB patients to access TB diagnostic tests.

At the health facilities, in addition to screening for TB in the outpatient and HIV clinics, nutrition clinics and inpatient wards should be prioritised by program managers as the two additional high yield areas. To further aid the diagnosis of TB in these two areas, additional diagnostic tools e.g., chest X-rays should be provided and healthcare workers should be trained in the clinical diagnosis of TB.

## Declarations

### Availability of data and materials

The datasets during and/or analyzed during the current study available from the corresponding author on request.
